# Particulate Matter (PM) and Parent, Nitrated and Oxygenated Polycyclic Aromatic Hydrocarbon (PAH) Emissions of Emulsified Heavy Fuel Oil in Marine Low-Speed Main Engine

**DOI:** 10.3390/toxics12060404

**Published:** 2024-05-31

**Authors:** Penghao Su, Hanzhe Zhang, Liming Peng, Lihong Zhu, Tie Li, Xiaojia Tang, Yimin Zhu

**Affiliations:** 1 College of Ocean Science and Engineering, Shanghai Maritime University, 1550 Haigang Ave, Shanghai 201306, China; 202230410033@stu.shmtu.edu.cn (H.Z.); plming317@163.com (L.P.); zlh980319@163.com (L.Z.); 2 International Joint Research Center for Persistent Toxic Substances (IJRC-PTS), Shanghai Maritime University, Shanghai 200135, China; 3 Department of Environmental Science and Engineering, Dalian Maritime University, Dalian 116026, China; listeel@dlmu.edu.cn (T.L.); xiaojiatang@dlmu.edu.cn (X.T.); ntp@dlmu.edu.cn (Y.Z.)

**Keywords:** emulsified HFO, ship stack exhaust, PM, PAH species

## Abstract

To understand the influences of emulsified fuel on ship exhaust emissions more comprehensively, the emissions of particulate matter (PM), nitrated, oxygenated and parent polycyclic aromatic hydrocarbons (PAHs) were studied on a ship main engine burning emulsified heavy fuel oil (EHFO) and heavy fuel oil (HFO) as a reference. The results demonstrate that EHFO (emulsified heavy fuel oil) exhibits notable abilities to significantly reduce emissions of particulate matter (PM) and low molecular weight PAHs (polycyclic aromatic hydrocarbons) in the gas phase, particularly showcasing maximum reductions of 13.99% and 40.5%, respectively. Nevertheless, burning EHFO could increase the emission of high molecular weight PAHs in fine particles and pose a consequent higher carcinogenic risk for individual particles. The total average (gaseous plus particulate) ΣBEQ of EHFO exhausts (41.5 μg/m^3^) was generally higher than that of HFO exhausts (18.7 μg/m^3^). Additionally, the combustion of EHFO (extra-heavy fuel oil) can significantly alter the emission quantity, composition, and particle-size distribution of PAH derivatives. These changes may be linked to molecular structures, such as zigzag configurations in C=O bonds. Our findings may favor the comprehensive environmental assessments on the onboard application of EHFO.

## 1. Introduction

Marine transportation has been the most important mode of transportation in international trade characterized by good economy and emission per unitized cargo. It is regulated or recommended by most of the coastal countries to switch highway and railway transports to shipping transport for economic and environmental sakes. As a result, shipping will share part of the emission burden of on-road traffic [1,2]. This study aims to tackle the challenge of reducing emissions from marine transportation, specifically focusing on particulate matter (PM) and polycyclic aromatic hydrocarbons (PAHs) emitted from emulsified heavy fuel oil (EHFO) used in marine low-speed main engines.

Oxides of nitrogen (NOx), along with oxides of sulfur (SOx) and particulate matter, constitute the primary pollutants in ship exhaust. According to [3], the sulphur content in fuel oil used on ships operating outside designated emission control areas is limited to 0.50% m/m (mass by mass), a significant reduction from the previous limit of 3.5%. The impact of these substances on the environment and human health cannot be ignored. The MARPOL convention sets requirements for nitrogen oxide (NOx) emissions. The NOx emission limits are to be implemented in three phases. The upcoming ‘Tier III’ emission standards, which are about to take effect, mandate a reduction of approximately 70% in emission limits compared to Tier II [4]. The IMO’s Tier III NOx emission standards are mandatory for all newly constructed ships operating in designated emission control areas (ECAs) after 1 January 2016. Outside of ECAs, known as non-ECA zones, ships are required to comply with the Tier I or Tier II standards, depending on the engine type and the year of construction. Depending on the catalyst utilized, SCR techniques encompass NH_3_-SCR, CO-SCR, HC-SCR, and H^2^-SCR and are a focal point of current discussions [5,6,7]. However, SCR systems necessitate precise operational conditions and are prone to challenges stemming from catalysts and reducing agent leakage. Additionally, the practice of slow steaming, aimed at increasing fuel efficiency, is not compatible with many SCR installations, as the lower temperatures in the exhaust gas are not conducive to SCR catalyst performance.

In addition to onboard control, there are multiple approaches to consider, such as replacing it with advanced diesel engines or changing the type of fuel used. There are numerous varieties of fuels available as alternatives to deal with the ship stack emission problems. Fuel blends obtained by adding water, microalgae biodiesel, methanol or butanol are proved to be efficient for emission reductions [8,9,10]. Water-emulsified fuels are practical and economical for NOx reduction compared to expensive large-scale denitrification facilities, such as SCR [11]. Studies on the onboard commercialization of the emulsified fuel are considered in accordance with the IMO’s (International Maritime Organization) increasing focus on NOx reduction [12,13,14]. 

On the other hand, modifying fuel characteristics can result in emission variations for various contaminants as well. For instance, compared to the neat fuel, burning an acetone–butanol–ethanol diesel blend can reduce PM emissions by 17.8–50.4% on a four-stroke single-cylinder engine [15], and burning waste cooking oil biodiesel can reduce CO and PM emissions by, respectively, 7–25% and 18–48% on a 4-cyclinder diesel engine [16]. 

Moreover, polycyclic aromatic hydrocarbons (PAHs) and their derivatives are a series of harmful compounds in ship stack exhausts [17]. They are of great concern due to their mutagenicity and carcinogenicity [18]. Our previous study has observed that burning the blends of biodiesel and marine gas oil on ship auxiliary engines nevertheless can reduce the bulk emission of PM and PAHs, concentrate the particulate PAHs, and enhance the toxicity of the particle matters [19]. Similar results are reported in the tests on waste edible oil [20], animal fat [21], Fischer–Tropsch [22] and sugarcane biodiesels [23] as well. Biomass fuel combustion generates PAH components mainly composed of naphthalene, accounting for almost half of the total, which is also the primary component of polycyclic aromatic hydrocarbons in mineral oil combustion [24,25]. In addition, the components of PAHs were also detected in the tail water of the desulfurization process [15]. Phenanthrene is one of the primary compounds of PAHs generated from the combustion of fossil fuels [26,27,28], it is necessary to make comprehensive assessments on the emissions before the onboard application of emulsified fuel. 

Furthermore, the concept of environmentally sustainable and eco-friendly maritime vessels has garnered considerable recognition, prompting a burgeoning body of research in this domain. However, an analysis of the 2020 International Maritime Organization’s (IMO) Data Collection System (DCS) statistics reveals that conventional heavy and light fuel oils continue to dominate marine energy consumption. This underscores the reality that, to date, alternative, cleaner energy sources have yet to supplant traditional fuels within the maritime industry [29].

The aim of this study is to reveal the effects of water emulsification with heavy fuel oil (HFO) on the emissions of PM and PAH species. Tests will be carried out on a bench with a slow-speed two-stroke marine engine. Both the engine and fuel are the most widely adopted by in-use ship propulsion systems. The PM and nitrated, oxygenated and parent PAHs will be particle size, segregated and monitored to figure out the effect mechanism preliminarily and provide data references for emulsified HFO emissions. Employing this methodology enables the precise monitoring and categorization of particulate matter (PM) and polycyclic aromatic hydrocarbons (PAHs) based on particle size. Such an approach yields initial understandings of the underlying mechanisms and contributes to a comprehensive dataset that is instrumental in shaping forthcoming regulations and driving innovation in the realm of marine emission mitigation.

## 2. Experimental Setup and Methods

### 2.1. Experimental Setup

The experiments were carried out on a 6-cylinder two-stroke diesel engine with a low speed of 142 rpm in the Engine Performance Test Lab of Shanghai Maritime University. The main specifications of the test diesel engine are listed in Table 1. The engine was designed to be representative of a typical low-speed marine engine with burning HFO, and the injection nozzle was not modified when burning EHFO to simulate the fuel changing onboard.

The experiments were conducted with commercial heavy fuel oil (HFO), which is a representative fuel for offshore marine main engines, and prepared emulsified heavy fuel oil (EHFO), which contained water at a rate of 5%vol and was verified to be able to decrease the emission of NO_x_ in a test carried on a high-speed diesel engine. We believe that it may be possible to produce EHFO onboard by substituting the purification process with clarification. This could be a significant advantage, as it would simplify the fuel preparation process and potentially reduce costs. Table 2 summarized the full parameters of the test fuels.

The engine loads ranged from idle to 75% and the test operation modes were 25%, 50% and 75% (the most common load point). To obtain reliable samples, the engine was operated for a few minutes trying to ensure that lubricating oil temperature, cooling temperature and exhaust gas temperature were in a steady state when sampling. In addition, the fuel consumption of the test fuels was reported in Table 3, showing that the engine operated relatively stable during the samplings.

### 2.2. Sampling Methods and Exhaust Measurements

The initial temperature of exhaust gas was ~350 °C at the sampling point on the stack 20 m downstream the engine exhaust outlet. The sampled exhaust gas was diluted with ambient air and then led the temperature fall below 50 °C for diluted gas. Particulate samples were collected from the diluted gas using an Anderson 8-stage sampler (Thermo-Andersen 20–800, United States) loaded with quartz fiber filters (QFF, 8.1 cm diameter, Munktell MK360, Sweden). The cutoff aerodynamic diameters for each stage were <0.43, 0.43–0.65, 0.65–1.1, 1.1–2.1, 2.1–3.3, 3.3–4.7, 4.7–5.8, 5.8–9.0 and >9.0 μm. Gaseous samples were collected by drawing air (from the outlet of the Anderson 8-stage sampler) through a chamber housing a layer of polyurethane foam plugs (PUF, 6.3 cm diameter and 4.0 cm thick, Tisch Environmental Inc., Maumee, OH, USA). The sampling time was 5 min, and the airflow rate was 28.3 L min^−1^ for each sampling.

Before sampling, the quartz fiber filter was baked in a muffle furnace for 2 h at 600 °C to remove background pollutants, and we recorded their own weight as initial weight [30]. The PUFs was pre-processed and cleaned with Soxhlet extraction with dichloromethane. Fuel consumptions were continuously measured during samplings and listed in Table 3.

### 2.3. Chemical Analysis Methods

The particulate samples on the quartz filter membranes were wrapped with tinfoil papers and dried in a desiccator for two days until constant weight. The difference of the constant weight and initial weight of membranes was the weight of PM. Then, each quartz filter membrane (containing particulate PAHs) was put in a glass sample bottle, spiked with 5 recovery surrogates of PAHs (naphthalene-d8, acenaphthene-d10, phenanthrene-d10, chrysened12 and perylene-d12) and 5 recovery surrogates of nitrated PAHs (nPAHs) and oxygenated PAHs (oPAHs) (nitronaphthalene-d7, nitroanthracene-d9, nitropyrene-d9, nitrochrysene-d11, anthraquinone-d8), then extracted three times using 20 mL of dichloromethane for 20 min in an ultrasonic bath. PUFs also needed to be wrapped in tinfoil papers and dried for a few days to remove the water. Then, the PUFs were spiked with the same recovery surrogates and extracted with Soxhlet extraction with 80 mL dichloromethane for 24 h. The extracts of PUFs were reduced to 2 mL under a gentle stream of nitrogen and then cleaned up using dispersive solid phase extraction. Finally, the extracted samples were spiked with a known amount of volumetric internal standards (hexamethylbenzene) and sent to instrumental analysis. 

PAHs were determined using an Agilent 7890-5975C gas chromatograph coupled with a mass selective detector (GC/MS) equipped with a DB-5 ms capillary column (15 m, 0.25 mm film thickness, Agilent, Santa Clara, CA, USA). Helium was used as the carrier gas at a constant flow of 2.0 mL min^−1^. The injection volume was 2.0 μL. The temperature of the injector, detector, transfer line and EI source was 280, 270, 280 and 230 °C, respectively. The column oven temperature program was 4 °C min^−1^ from 60 to 300 °C. Identification of each individual PAH was based on its retention time and the specific primary ion fragment *m*/*z* of a corresponding authentic standard.

Negative chemical ionization (NCI) using methane ionization gas (40 mL min^−1^) and a source temperature of 200 °C was employed for analysing these nPAHs and oPAHs. The detected compounds have been listed in Table 4 and Table 5, along with their retention time (RT) and characteristic ion. The oven temperature program analysis was 40 °C (held 1.7 min), ramped to 150 °C at 20 °C min^−1^ and held for 10 min, ramped to 220 °C at 10 °C min^−1^ and held for 10 min and finally ramped to 310 °C and held for 15 min. Abundances three times higher than the base peak were detected.

A spiked blank and a reagent blank sample were prepared to assess the recovery of the analytical method. Recoveries of surrogate compounds ranged between 75.2% and 113%. Analyzed reagent blank samples were found to contain undetectable amounts of any interference. The limit of quantification (LOQ) was determined from the standard deviation by analyzing ten blank samples. LOQ was estimated as the mean blank value plus ten times the standard deviation. The LOQs of PAHs in the exhaust and fuels were 0.01 μg/Nm^3^ and 0.01 μg mL^−1^ for exhausts and fuels, respectively. The final sample concentrations were surrogate recovery corrected.

Same instrumental analysis and QA/QC processes were used in our previous study [31]. In this test, recoveries of surrogate compounds ranged between 75.2% and 113%. The final sample concentrations were surrogate recovery corrected.

### 2.4. Calculation of Emission Factors

The exhaust flow rates for the engine were calculated using the carbon balance method specified in ISO 8178-2 [32], assuming complete conversion of fuel carbon to CO_2_. Based on the fuel consumptions (Table 3) and pollutant concentrations, the emission factors (EFs) of PM and PAHs were determined using similar methodology in our previous study [33,34].

Emission data are reported as power-based emission factors, which can be calculated from the measured pollutant concentrations using the following equations:

(1) $$V_{E}=\frac{C\%\times FC}{M}\times R÷{CO}_{2}\%$$

(2) $${EF}_{PM}={∆m}_{PM}÷V_{S}\times V_{E}$$

(3) $${EF}_{PAH}={∁}_{PAH}÷V_{S}\times V_{E}$$

where *V_E_* is the emission volume, *V_S_* is the sampling volume and *M* is the molecular weight of carbon, *FC* is the fuel consumption, *R* is the gas constant and Δ*m* is the difference of filter mass before and after sampling in the Anderson eight-stage cascade impactor (TE-10-800, TISCH, Maumee, OH, USA). The constant air sampling flow rate is 28.31 L min^−1^, pumping for 5 min under each operating mode, and the sampling volume is 142 L.

## 3. Results and Discussion

### 3.1. Effects on Emission Characteristics of PM and p,n,o-PAHs

#### 3.1.1. Effects on Emission Characteristics of PM and PAHs

In this test, the *EF*s of PM and PAHs when burning HFO ranged from 510 to 738 mg kWh^−1^ and 1153 to 2482 µg kWh^−1^ (Table S1), varying with the operation modes. These results were comparable to those reported in the literature on HFO emissions (Figure S2). By comparison, the EF_PM_ and EF_PAHs_ the *EF*s of burning EHFO ranged from 505 to 635 mg kWh^−1^ and 1037 to 1486 µg kWh^−1^, respectively. The reductions derived from emulsification could also be observed in previous investigations [35,36,37], especially for PAHs. Unfortunately, we were unable to compare the reduction effects of emissions with other alternative ship fuels, such as dual fuel (DF), marine diesel oil (MDO) and biodiesel, because of limited monitoring capabilities on ship main engines (Table S1).

**Figure 1:**
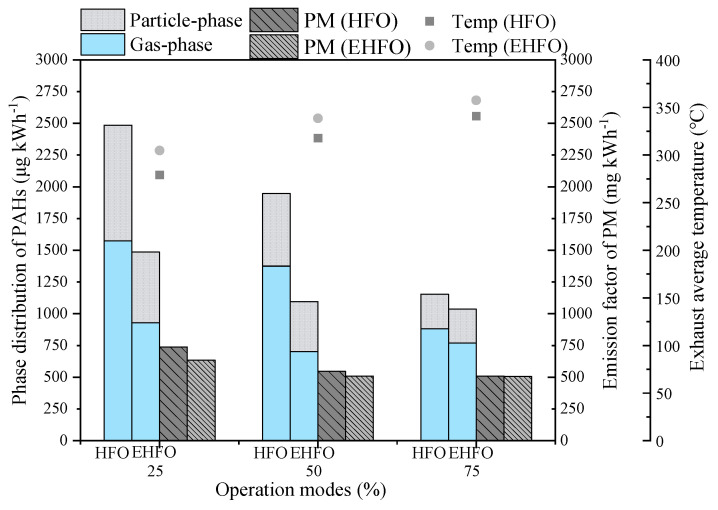
EFs under various operation modes.

A further analysis illustrated that burning EHFO resulted in greater reductions in PM and PAH emissions at lower loads (Figure 1). At the load of 25%, emulsification could reduce 13.9% and 40.2% of PM and PAH emissions, respectively. By contrast, at the load of 75%, the reductions in PM and PAH emissions were 0.85% and 10.0%, respectively. Furthermore, burning EHFO could result in a slightly greater reduction in gaseous ∑_16_PAHs compared to particulate PAHs, i.e., the gaseous and particulate PAHs was reduced by 40.9%, 48.9% and 12.6% and 40.2%, 43.7% and 10.0%, respectively, at the loads of 25%, 50% and 75%.

The reductions in *EF*_PM_ and *EF*_PAHs_ by burning EHFO could be partly attributed to the higher combustion temperature in cylinders, which was reflected in a higher combustion temperature (Figure 1). Both pyrolysis and oxidation rates of PAHs (who are the precursors of PM as well) can increase with combustion temperature while the oxidation rate increases faster so that less PAHs and PM survived [38]. It is why *EF*_PM_ and *EF*_PAHs_ decreased with the increases in the power output and the exhaust temperature as well.

To contextualize our findings within the broader scientific discourse, we compared our results with those from similar studies. For instance, the reduction in PM emissions at lower loads aligns with findings by [39]. However, our study extends the understanding by detailing the impact of EHFO at various load levels, a dimension that has not been extensively explored in the literature.

Furthermore, under the combustion temperature in diesel cylinders (normally 400–1600 K, Table S2), water can be atomized and disrupts fuel droplets into finer sizes (namely the ‘micro-explosion effect’), which can enhance the mixing rate and combustion rate [40]. Water atomization can also generate more oxidative reactants, such as OH radicals, to promote the oxidation reactions of PAHs and PM [41], thus resulting in less emissions [42,43]. 

With respect to the possible opposite influence of burning EHFO, i.e., water evaporation will delay the maximum cylinder pressure point and lower engine efficiency and therefore result in more emissions [44], it was unlikely the case for the tests in this study.

#### 3.1.2. Effects on Emission Characteristics of n,o-PAHs

Nine species and eight species among the twenty-two monitored species of n, o-PAHs were detected in the HFO and EHFO exhausts, respectively. The total emissions (sum of gaseous and particulate phases) of nPAHs burning HFO and EHFO were 20.2, 6.50 and 2.30 μg kWh^−1^ and 7.20, 7.31 and 10.0 μg kWh^−1^, respectively, at three modes (Figure S3). Compared with burning HFO, the nPAH emissions when burning EHFO were decreased by 65%. 1N-PYR, the predominant nPAH species in the NIST diesel SRM 1975 [45] and real-world diesel exhausts [17,46], was abundant in particles of HFO and EHFO exhausts as well.

The relative abundance could be observed for oPAHs compared to nPAHs. The total emissions (sum of gaseous and particulate phases) of oPAHs emitted from HFO and EHFO were 54.8, 38.2 and 17.1 μg kWh^−1^ and 87.4, 96.6 and 79.2 μg kWh^−1^, respectively, at three modes. Representative oPAH species of diesel exhausts, such as 9,10-ATQ, BZO and BD [46,47], were detected, as well as 9-FO. oPAHs detected were mostly on particles, which can be explained by a newly proposed pathway for oPAH formation in the combustion process: PAH → incipient soot → oPAH formation → selective thermal decomposition of oPAH [37]. 

Emulsification and a likely consequent OH radical supplement affect the emissions of oPAHs dramatically. In theory, both the formation and decomposition of oPAHs are oxidation reactions [37,48,49], and therefore, the effect of the oxidizer on the final emission is dependent on the additive oxidizers. For example, in practice, adding biodiesel in diesel can lead to increases in oPAH emissions due to excess oxygen in biodiesel, with a half biodiesel addition leading to the highest increase, while pure biodiesel presents a smaller increase [46]. In the present tests, there was additive water with an amount that lead to a reduction in 9-FO emissions by 70% while it increased in 9,10-ATQ, BD and especially BZO emissions. It was reasonable to assume that the formations of all the oPAHs were promoted due to the additional OH radicals and higher combustion temperature when burning EHFO. Accordingly, the different effects on final emissions of individual oPAH species were derived by the process of OH oxidation. 9,10-ATQ, BD and BZO are the most stable oPAH species [37], and thus, additional OH radicals did not consume all the additional formations. In particular, BZO has three benzene ‘branches’ near the zigzag cite of the C=O bond and a non-symmetrical structure (Figure S4), which might be responsible for its pretty survival abilities from OH radical oxidations.

### 3.2. Effects on PAH Composition and Phase Distribution

**Figure 2:**
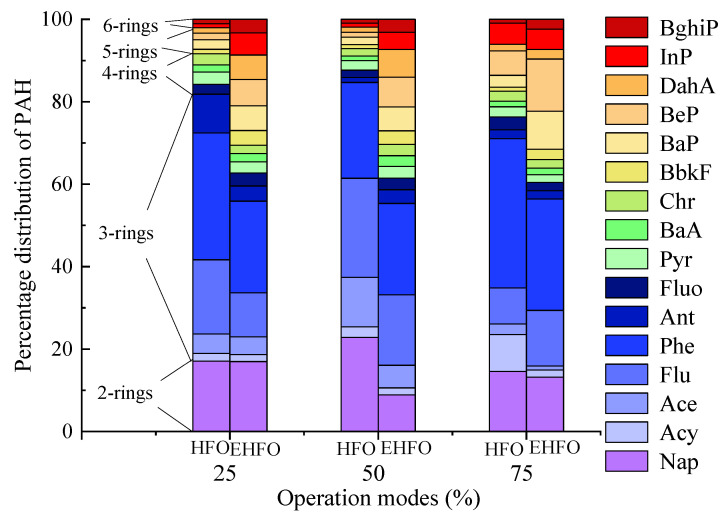
The distribution of PAHs in the sum of the particle phase and gas phase.

Figure 2 showed that Nap, Flu and Phe were the most abundant PAHs species, accounting for 182%, 17.0% and 30.1% and 13.0%, 13.8% and 23.8% in HFO and EHFO exhaust, respectively (an average under all loads). This was like the onboard observations on HFO exhausts [50,51,52,53,54]. Nevertheless, a previous test on the same bench of this study has observed that Fluo is the predominant PAH compound [52] for unknown reasons.

It was regular to observe that over 70% of LMW-PAHs (low molecular weight PAHs, 2, 3, 4-ring) were emitted in gas phase, and on the contrary, most of the HMW-PAHs (high molecular weight PAHs, 5, 6-ring) were emitted with particles. Accordingly, the variation in gaseous and particulate emissions will dominate the total emissions of LMW- and HMW-PAHs, respectively. Figure S5 showed that burning EHFO could reduce the emissions of LMW-PAHs in the gas phase (by 42.2%, an average under all loads) and nevertheless enhance the emissions of HMW-PAHs in the particle phase (by 112%, an average under all loads). Consequently, the total emissions of LMW-PAHs and HMW-PAHs were reduced and enhanced by 54.9%, 57.8% and 28.1% and 118%, 140% and 75.0%, respectively, at three loads.

The chemical converting trend from LMW- to HMW-PAHs in EHFO exhausts, as shown in Figure 2, could be explained by the generative mechanisms of PAHs in internal combustion cylinders. As revealed using the laser-induced fluorescence method, light PAHs are first formed just after the premixed heat release peak, followed by a consequent temporal growth of PAH molecular size [55]. In other words, the heavy PAHs form via the pyrosynthesis process [56]. In this aspect, when burning EHFO, a high cylinder temperature (reflected in a higher exhaust temperature, as shown in Figure 1) and a consequent high cylinder pressure would promote the collisions of low molecular weight aromatic compounds and the formations of heavy PAHs.

In addition, for light PAHs, such as Nap, Ace, Flu and Phe, the oxidations by OH radicals from water atomization play an important role on reducing their emissions when burning EHFO. The temperature in the oxidation zone (in the post-flame region) in this tested engine could be valued as ~1400 K based on the measured cylinder pressure and model [57]. At 1400 K, the oxidative activity of OH radicals (*k =* 4.70 × 10^−12^ cm^3^ molecule^−1^ s^−1^, calculations were listed in Table 6) is as strong as O radicals (*k =* 7.26 × 10^−12^ cm^3^ molecule^−1^ s^−1^), which serve as the predominant oxidant. This means that OH radicals can promote the oxidations of light PAHs if they are formed. By contrast, the oxidations of heavy PAHs by OH or O radicals are not such effective, and the mechanisms are not yet available.

As a result, less LMW-PAHs remained, while on the contrary, more HMW-PAHs were emitted in EHFO exhaust. Moreover, it was worth noting that the emission of gaseous Nap was reduced by 183 μg kWh^−1^ when burning EHFO, ranking at the top in quantitative reductions among all PAH compounds. As Nap has been observed as the primary pollutant (within the scope of 16 EPA priority PAHs) in ship stack exhausts and the surrounding environments [31,54,58,59], burring EHFO may be meaningful to emission reductions of PAHs. 

### 3.3. Effects on Size Distributions of PM and p,n,o-PAHs

Figure S7 showed that particles were apt to distribute in fine size in both HFO and EHFO exhausts. This distribution consisted with typical nucleation patterns in combustion exhausts [60], including real-world ship engine exhausts [61,62]. Compared to HFO, although the total *EF*_PM_ was reduced, the emissions of fine particles (*D*_P_ < 0.65 μm) were enhanced by burning EHFO, especially at 50% and 75% loads, which might pose a higher risk of inhalation of exhausted particles (without concerning the toxicity of individual particle herein).

**Figure 3:**
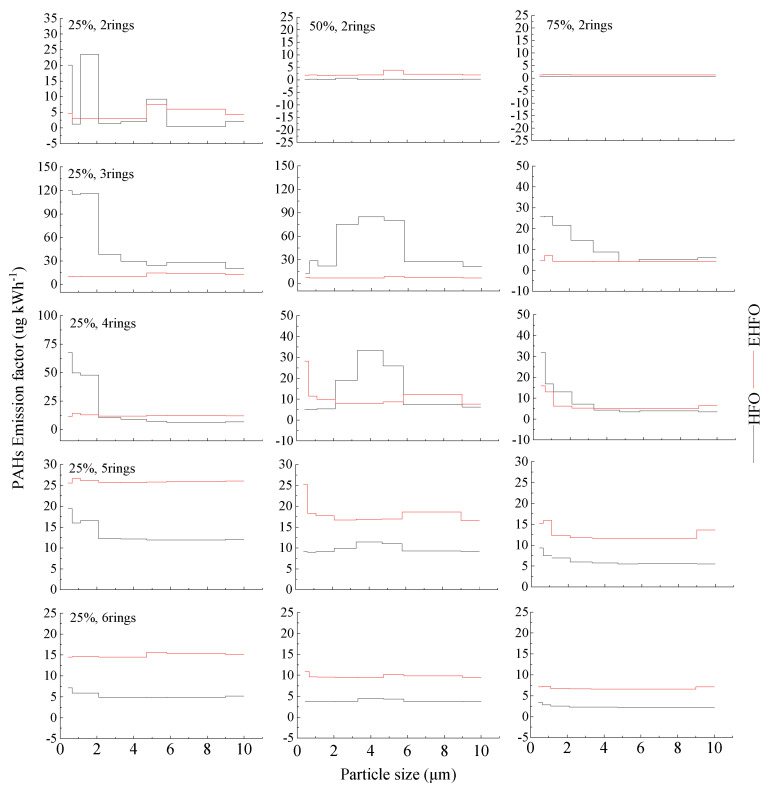
Particle-size distributions of PAHs.

Figure 3 plotted that the particulate PAHs mostly peaked in fine sizes in the HFO exhausts, especially at the load of 25%. By comparison, emulsification could weaken the peak in fine sizes for LMW-PAHs and enhance the emissions of HMW-PAHs in all sizes of particles. The size distribution of PAHs in HFO and EHFO exhausts both deviated from the equilibrium distribution rule in ambient air, i.e., LMW- and HMW-PAHs affiliated with coarse and fine particles, respectively [53,63].

To provide a first approximation of a health risk assessment on particulate PAHs, the size distribution of PAHs was further plotted in terms of the concentrations in per unit particles (PAH_perPM_, mg g^−1^, Figure S6). Notable effects of emulsification on PAH_perPM_ could be observed at the load of 25%, including the following: obvious PAH_perPM_ peaks appeared in the particles with *D*_P_ of 2.1–3.3 μm; the concentrations in per unit particles for LMW-PAHs were reduced in PM_2.1._ The valley in particle-size distribution at a *D*_P_ of 2.1–3.3 μm (Figure S7) could be responsible for the PAH_perPM_ peaks therein, while the dramatic decrease in particulate LMW-PAHs (Figure 3) overwhelmed the decrease in PM_2.1_ (Figure S7) and resulted in LMW-PAH_perPM_ reductions in PM_2.1_. In addition, the HMW-PAHs in per unit EHFO particles were enriched at all test loads, which implies a higher health risk for each single EHFO particle.

**Figure 4:**
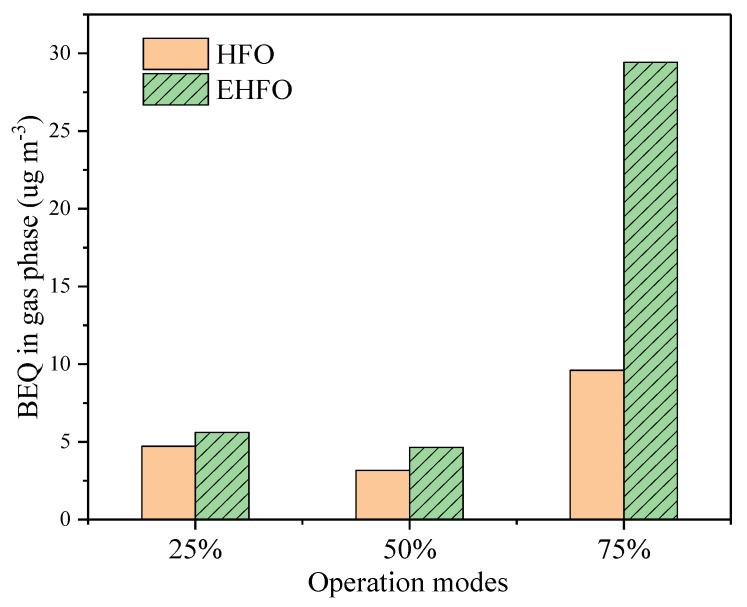
The Σ*BEQ* in gas phase.

Burning EHFO dramatically reduced 9-FO in fine particles and resulted in a reduction in total emissions at three loads (Figure 4). By contrast, EHFO increased 1N-PYR in all particles at the loads of 50% and 75%. With respect to oPAHs, EHFO increased their emissions in all particles, especially in finer ones, at all loads.

### 3.4. Carcinogenic Properties

The toxic equivalent factors (TEFs) method was considered to be a more common and reliable method to identify and evaluate the carcinogenicity of PAHs [64,65,66]. BaP had the highest carcinogenicity and usually was used as a reference compound. The BaP equivalent (BEQ) is calculated from the individual concentration in each gaseous and particulate sample and the TEFs of individual target compounds using Equation (4). Table S6 shows the TEF values of 15 PAHs and 1N-PYR [67,68]. The TEFs of other PAH derivates were not available, so the toxicity was not calculated in ∑BEQ.

(4) $$\sum BEQ=\sum_{i}^{n=1}(C_{i}\times {TEF}_{i})$$

where *C*_i_ is the concentration of the *i*th target compound (ng m^−3^) and *TEF*_i_ is the toxic equivalency factor of the *i*th target compound. The ∑BEQ in gas and particles were calculated and showed in Figure 4 and Figure S8, respectively.

Unfortunately, the total average (gaseous plus particulate) Σ*BEQ* of EHFO exhausts (41.5 μg m^−3^) were generally higher than those of HFO exhaust (18.7 μg m^−3^) due to the increase in HWM PAHs (Bap, DahA) (Figure S5). This indicated that EHFO exhaust might pose higher carcinogenic risks than HFO exhausts despite the emission reductions. Other than that, no notable Σ*BEQ* alternation derived from burning EHFO was observed. The gas–particle distribution and particle-size distribution of *BEQ* were similar between HFO and EHFO exhausts.

## 4. Conclusions

Water emulsification plays a role in altering the emission of PM, PAHs and their derivatives. Burning EHFO could reduce the emissions of PM and LMW-PAHs in the gas phase, with a respective maximum reduction of 13.9% and 40.5% at low operation mode. Nevertheless, burning EHFO could increase the emission of HMW-PAHs in fine particles and pose a consequently higher carcinogenic risk to ecology. Burning EHFO could reduce the nPAH emissions while increasing the oPAH emissions. In addition, the compositions of the nine detected species of PAH derivatives have been altered dramatically, which could be explained by the addition of OH radicals by burning emulsified fuel and the difference in the molecular structure of the derivatives. These findings are valuable for assessing the advantages and disadvantages of the onboard application EHFO. 

This study differs from the previous literature on denitrification and fuel-saving methods, providing significant reference values for a comprehensive assessment of emulsified oil and the design of exhaust gas treatment devices, such as the PAH component in the tail water of desulfurization towers. Furthermore, this study presents an exhaustive evaluation of emulsified heavy oil technology, establishing a foundational dataset for comparative analyses in subsequent investigations concerning alternative fuel mixtures and biodiesel applications.

## Figures and Tables

**Table 1:** Research engine specifications.

Engine Mode	6S35ME-B9
Type	6-cylinder, two strokes
Bore/stroke	350/1550 mm
Revolutions per minute	142 rpm
Max continuous output	3570 kW

**Table 2:** Fuel properties of HFO and EHFO.

Parameter	HFO	EHFO
Density at 15 °C, kg/m^3^	976	982
Viscosity (CST) at 50 °C	161	159
Water content, % (*v*/*v*)	0.32	4.15
Flash Point (Close), °C	81.0	63.0
Carbon content, wt.%	86.17	82.85
Hydrogen content, wt.%	13.01	12.94
Oxygen content, wt.%	0.68	4.07
Lower heating value, kJ/g	42.56	39.00

**Table 3:** Fuel consumption, g kWh^−1^.

Operation Modes	25%	50%	75%
HFO	211.9	237.4	180.4
EHFO	227.0	219.7	202.6

**Table 4:** GC retention time (RT) and characteristic ion (T) of N-PAHs.

N-PAHs	RT	T	N-PAHs	RT	T
1-Nitronaphthalene	8.995	173,174	3-Nitrophenanthrene	16	223,224
2-Nitronaphthalene	9.374	173,174	2-Nitroanthracene	16.804	223,224
2-Nitrobiphenyl	11.054	199,200	3-Nitrofluoranthene	22.293	247,248
3-Nitrobiphenyl	12.446	199,200	1-Nitropyrene	23.253	247,248
5-Nitroacenaphthene	12.859	199,200	7Nitrobenzo(a)anthracene	25.926	273,274
2-Nitrofluorene	13.909	211,212	6-Nitrochrysene	26.836	273,274
9-Nitroanthracene	14.314	223,224	6-Nitrobenzo[a]pyrene	29.624	297,298
9-Nitrophenanthrene	15.292	223,224			

**Table 5:** GC retention time (RT) and characteristic ion (T) of O- PAHs.

O-PAHs	RT	T
Naphthalene-1-alsehyde	8.058	156,157
9-Fluorenone	10.259	180,181
9-Formylphenanthrene	14.06	206,207
9,10-Anthraquinone	12.454	208,209
1,4-Anthraquinone	13.126	208,209
Benzanthrone	21.097	230,231
Benz(a)anthracene-7,12-dione	23.024	258,259

**Table 6:** Reaction coefficient k of benzene oxidations by OH and O radicals.

Reactions	*k*	*T* (K)
$OH+\left[-C_{6}H_{6}-\right]\to H_{2}O+\left[-C_{6}H_{5}-\right]$	2.7 × 10^−16^*T*^1.42^exp(−732/*T*)	400–1495
$O+\left[-C_{6}H_{6}-\right]\left(+M\right)\;\to \;\;\left[-C_{6}H_{5}-\right]-OH\left(+M\right)$	3.7 × 10^−11^exp(−2280/*T*)	298–1400

The oxidation rates by OH and O radicals of Nap, Phe and Pyr are equivalent to benzene (Hai and Frenklach, 1997).

## Data Availability

Data is contained within supplementary material in Tables S2–S7.

## Supplementary Materials

The following supporting information can be downloaded at: https://www.mdpi.com/article/10.3390/toxics12060404/s1, Figure S1: The distribution of PAH compounds in gaseous and particle phase; Figure S2: EFs of PM and PAHs in this study and references; Figure S3: EFs of nPAHs and oPAHs; Figure S4: The molecular structure of oPAHs; Figure S5: The difference in EFs between EHFO and HFO; Figure S6: Particle-size distributions of PAHperPM; Figure S7: Size distribution of PM; Figure S8: The ∑BEQ in the particle phase. Table S1: EFs in the previous studies (mg kWh^−1^, μg kWh^−1^); Table S2: The correlation between the combustion temperature and pressure in the cylinder of the model; Table S3: Ratios of PAH emission factors between EHFO and HFO; Table S4: Emission factors of detected PAHs, μg kWh^−1^; Table S5: EFs of n,o-PAHs, μg kWh^−1^; Table S6: Toxic Equivalency Factors (TEF) for individual PAHs and 1-Nitropyrene (TEFs of n, oPAHs, except for 1-Nitropyrene, are not avilable at present);Table S7: The BaP equivalent (BEQ) of 15 PAHs and 1-Nitropyrene (ng m^−3^) [69,70,71,72,73,74].
