# Supplementary material for: Particulate Matter (PM) and Parent, Nitrated and Oxygenated Polycyclic Aromatic Hydrocarbon (PAH) Emissions of Emulsified Heavy Fuel Oil in Marine Low-Speed Main Engine
Source: Toxics. 2024 May 31;12(6):404. doi: 10.3390/toxics12060404 (PMC11209265; doi:10.3390/toxics12060404)
Supplement: Supplementary file 1 [file toxics-12-00404-s001.zip › toxics-3000194-supplementary.pdf]

Supplementary Materials: Particulate Matter (PM) and Parent, Nitrated and Oxygenated Polycyclic Aromatic Hydrocarbon (PAH) Emissions of Emulsified Heavy Fuel Oil in Marine Low-Speed Main Engine

Text 1. Instrumental analysis and QA/QC

The 16 USEPA priority PAHs were determined in this study: naphthalene (Nap), acenaphthene (Ace), acenaphthylene (Acy), fluorene (Flu), phenanthrene (Phe), anthracene (Ant), fluoranthene (Fla), pyrene (Pyr), benzo(a)anthracene (BaA), chrysene (Chr), benzo(b)fluoranthene (BbF), benzo(k)fluoranthene (BkF), benzo(a)pyrene (BaP), dibenzo(a,h)anthracene (DBahA), indeno(1,2,3-cd)pyrene (InP) and benzo(g,h,i)-perylene (BghiP).

Fifteen nPAHs and seven oPAHs were also determined in this study: 1-nitronaphthalene (1N-NAP), 2-nitronaphthalene (2N-NAP), 2-nitrobiphenyl (2N-BPH), 3-Nitrobiphenyl (3N-BPH), 5-nitroacenaphthene (5N-ANA), 2-nitrofluorene (2N-FLU), 9-nitroanthracene (9N-ANT), 9-nitrophenanthrene (9N-PHE), 3-nitrophenanthrene (3N-PHE), 2-nitroanthracene (2N-ANT), 3-nitrofluoranthene (3N-FLT), 1-nitropyrene (1N-PYR), 7-nitrobenzo(a)anthracene (7N-BaA), 6-nitrochrysene (6N-CHR), 6-nitrobenzo[a]pyrene (6N-BaP) and naphthalene-1-alsehyde (1-NALD), 9-fluorenone (9-FO), 9-formylphenanthrene (PHE-9-ALD), 9,10-anthraquinone (9,10-ATQ), 1,4-anthraquinone (1,4-ATQ), benzanthrone (BZO) and benz(a)anthracene-7,12-dione (BD).

Table S1. EFs in the previous studies (mg kWh^{-1}, μg kWh^{-1}).

Fuel， engine type	PM	PAH	References
HFO, four-stroke, 37680kW, 1500rpm, 25%	1902	n.a.	(Sippula, Stengel et al. 2014)
HFO, two-stroke, middle speed	3100	n.a.	(Van der Gon and Hulskotte 2010)
HFO, four-stroke, middle speed	6500	n.a.	(Van der Gon and Hulskotte 2010)
HFO, four-stroke, 37680kW, 1500rpm, 25%	1902	n.a.	(Sippula, Stengel et al. 2014)
HFO, four-stroke, 37680kW,1500rpm, 50%	728	n.a.	(Sippula, Stengel et al. 2014)
HFO, 37680kW, 1500rpm, four stroke, 75%	843	n.a.	(Sippula, Stengel et al. 2014)
HFO, 54860kw	1360-1970		(Sax and Alexis 2007)
HFO, two-stroke, 50270kw, 104rpm	1091-1957	n.a.	(Agrawal, Malloy et al. 2008)
HFO, two-stroke, 36740kW, 102rpm	2000	n.a.	(Khan, Ranganathan et al. 2012)
HFO, 68530kW, 97rpm	1490	n.a.	(Khan, Ranganathan et al. 2012)
DF, four-stroke, 75%	154-236	n.a.	(Sippula, Stengel et al. 2014)
DO, 700kw, 1200rpm	1610-2570	n.a.	(Zhang, Chen et al. 2015)
DO, 3200kw, 900rpm	70-210	n.a.	(Zhang, Chen et al. 2015)
DO, 600kw, 1000rpm	30-50	n.a.	(Zhang, Chen et al. 2015)
MDO, two-stroke	2100	n.a.	(Van der Gon and Hulskotte 2010)
MDO, four-stroke	2200	n.a.	(Van der Gon and Hulskotte 2010)
DF, four-stroke	184-217	n.a.	(Sippula, Stengel et al. 2014)
Biodiesel, 400kw, 750rpm	423-1009	n.a.	(Petzold, Lauer et al. 2011)
HFO, four-stroke, 50%	n.a.	255	(Zhang, Chen et al. 2015)
HFO, four-stroke, 75%	n.a.	438	(Zhang, Chen et al. 2015)
HFO, 8850kw	n.a.	1313.2	(Zhao, Zhang et al. 2020)
HFO, 12492kw	n.a.	4080.7	(Zhao, Zhang et al. 2020)
HFO, 15534kw	n.a.	5887.3	(Zhao, Zhang et al. 2020)
HFO, 3639kw	n.a.	3635	(Zhao, Zhang et al. 2020)
DF, four-stroke	n.a.	40	(Sippula, Stengel et al. 2014)
MDO, 3456kw	n.a.	108.9	(Zhao, Zhang et al. 2020)
HFO, 3570kW, two-stroke, 25%	738	2483	This study
HFO, 3570kW, two-stroke, 50%	546	1946	This study
HFO, 3570kW, two-stroke, 75%	510	1153	This study
EHFO, 3570kW, two-stroke, 25%	635	1486	This study
EHFO, 3570kW, two-stroke, 50%	510	1095	This study
EHFO, 3570kW, two-stroke, 75%	505	1037	This study

HFO: heavy fuel oil;
EHFO: emulsified heavy fuel oil;
n.a.: not available.

Table S2. The correlation between the combustion temperature and pressure in the cylinder of the model (Yu, Duan et al. 2015). .

Crank Angle (deg)	Mean Temperature (degC)	Mean Pressure (MPa)
215	26	0.1
281	120	0.5
347	500	4.5
363	1600	12

deg: degree of the crank angle.
degC : degree Celsius.

Table S3. Ratios of PAH emission factors between EHFO and HFO

Engine load		25%	50%	75%
	LMW	0.51	0.43	0.77
	HMW	1.88	2.28	1.38
Gaseous	∑PAHs	0.59	0.51	0.87
	LMW	0.23	0.21	0.41
Particulate	HMW	1.18	1.47	1.41

	∑PAHs	0.61	0.69	0.98
	LMW	0.43	0.38	0.72
	HMW	1.32	1.65	1.39
Total	∑PAHs	0.60	0.56	0.90

LMW: low molecular weight;

HMW: high molecular weight.

Table S4. Emission factors of detected PAHs, μg kWh^{-1}

		EHFO			HFO		
Loads		25%	50%	75%	25%	50%	75%
Gaseous	Nap	217	79.9	126	365	442	164
	Acy	6.23	6.17	9.83	36.7	42.7	98.8
	Ace	55.5	55.4	7.16	108	227	26.5
	Flu	152	184	265	335	374	99.2
	Phe	293	221	138	424	222	321
	Ant	32.6	22.1	12.1	214	8.64	18.3
	Fluo	22.9	12.3	8.74	16.6	10.3	11.1
	Pyr	15.3	8.31	5.35	13.2	11.4	9.16
	BaA	4.65	4.26	1.73	5.25	3.27	3.39
	Chr	3.90	1.35	0.94	5.25	4.39	0.70
	BbkF	2.51	1.92	2.00	3.16	2.24	0.77
	BaP	16.4	10.8	58.1	18.9	9.77	14.5
	BeP	54.7	49.0	110	18.0	9.32	59.2
	DahA	42.8	43.8	2.93	3.97	2.32	5.27
	Inp	9.60	1.14	20.2	4.41	3.49	49.2
	BghiP	n.d	0.8	1	3.45	2.23	0.38
	∑PAHs	929	703	770	1575	1375	882
Particulate	NaP	34.2	17.5	10.3	59.6	1.67	4.08
	Acy	18.5	12.4	8.35	9.45	6.75	4.21
	Ace	8.47	4.75	2.78	8.18	7.27	2.71
	Flu	6.70	3.19	1.56	114	93.4	1.60

Phe	35.3	21.4	15.3	339	231	96.9
Ant	21.8	14.3	8.94	20.3	14.3	6.39
Fluo	23.3	18.5	12.0	42.3	24.1	25.2
Pyr	25.0	22.7	14.4	61.6	34.3	19.1
BaA	25.3	24.5	15.1	37.7	18.4	12.3
Chr	25.5	28.5	20.2	62.5	30.6	27.4
BbkF	50.5	34.3	24.1	23.1	16.4	10.8
BaP	71.8	52.5	37.5	39.4	26.0	18.4
BeP	39.8	30.1	21.2	20.6	13.9	9.39
DahA	45.2	30.0	21.0	28.9	21.6	13.3
Inp	70.3	44.7	31.1	20.4	14.8	9.30
BghiP	48.3	33.5	23.6	23.3	16.5	10.8
∑PAHs	557	393	267	910	571	272

Table S5. EFs of n,o-PAHs, μg kWh^{-1}

		EHFO			HFO		
loads		25%	50%	75%	25%	50%	75%
Gaseous	1N-NAP	0.35	0.22	0.25	0.24	0.09	0.04
	2N-NAP	0.23	0.26	n.35	0.67	0.12	0.40
	3N-BPH	0.06	0.06	0.28	1.31	0.81	0.03
	9N-ANT	0.18	0.18	0.20	0.50	0.09	0.10
	1N-PYR	0.29	0.34	0.42	6.68	0.17	0.02
	9-FO	0.89	1.82	2.17	3.88	0.64	3.6
	9.10-ATQ	0.67	0.98	1.36	8.33	4.56	0.50
	BZO	0.07	0.45	0.08	7.44	1.59	0.03
	BD	0.43	0.29	0.23	0.68	0.26	0.01
	∑nPAHs	1.11	0.80	1.5	9.40	1.28	0.58
	∑oPAHs	2.06	3.54	3.84	20.3	7.05	4.18
particulate	1N-NAP	1.60	1.6	1.60	0.67	0.58	0.11
	2N-NAP	n.d.	n.d.	n.d.	0.95	1.25	0.43
	3N-BPH	0.64	1.02	3.12	3.01	0.92	0.61
	9N-ANT	1.52	1.57	1.48	3.77	1.16	0.11
	1N-PYR	2.34	2.32	2.33	2.44	1.27	0.44
	9-FO	2.98	5.21	4.76	20.7	21.6	9.94
	9.10-ATQ	4.12	7.56	5.11	2.62	2.75	2.45
	BZO	73.4	73.4	60.3	9.70	5.78	0.22
	BD	4.85	6.95	5.11	1.39	1.02	0.29
	∑nPAHs	6.09	6.51	8.53	10.8	5.17	1.70
	∑oPAHs	85.3	93.1	75.3	34.4	31.1	12.9

n.d.: not detected.

Table S6. Toxic Equivalency Factors (*TEF*) for individual PAHs (Nisbet 1992) and 1-Nitropyrene (J, Collins et al. 1998) (*TEF*s of n, oPAHs, except for 1-Nitropyrene, are not avilable at present).

**PAHs and nitro-PAH**	***TEF***
Napthalene	0.001
Acenaphthylene	0.001
Acenaphthene	0.001
Fluorene	0.001
Phenanthrene	0.001
Anthracene	0.01
Fluoranthene	0.001
Pyrene	0.001
Benzo[a]anthracene	0.1
Chrysene	0.01
Benzo[b,k]fluoranthene	0.1
Benzo[a]pyrene	1
Indeno[1,2,3-cd]pyrene	0.1
Dibenzo[a,h]anthracene	5
Benzo[g,h,i]perylene	0.01
1-Nitropyrene	0.1

Table S7. The BaP equivalent (BEQ) of 15 PAHs and 1-Nitropyrene (ng m^{-3}).

	**HFO**						EHFO					
	**25%**		**50%**		**75%**		25%		50%		75%	
	**gas**	**particle**	**gas**	**particle**	**gas**	particle	Gas	particle	gas	particle	gas	particle
Nap	**59.4**	9.7	**95.4**	0.4	**59.4**	0.1	36.7	5.8	20.8	4.6	45.5	3.7
Acy	6.0	1.5	9.2	1.5	35.7	1.4	1.1	3.1	1.6	3.2	3.5	3.0
Ace	17.6	1.3	48.9	1.6	9.6	0.9	9.4	1.4	14.4	1.2	2.6	1.0
Flu	54.5	18.5	80.7	20.1	35.9	0.5	25.6	1.1	47.9	0.8	95.4	0.6
Phe	69.0	55.3	47.8	49.8	116.0	34.9	49.7	6.0	57.7	5.6	49.8	5.5
Ant	347.8	33.0	18.6	30.9	66.0	21.8	55.2	36.9	57.6	37.2	43.6	32.2
Fluo	2.7	6.9	2.2	5.2	4.0	9.0	3.9	3.9	3.2	4.8	3.1	4.3
Pyr	2.1	10.0	2.4	7.4	3.3	6.8	2.6	4.3	2.2	5.9	1.9	5.2
**BaA**	**85.5**	**613.6**	**70.5**	**395.7**	**122.7**	**430.5**	**78.7**	**453.8**	**110.9**	**637.0**	**62.3**	**543.9**
Chr	8.5	101.7	9.5	65.9	2.5	98.1	6.6	46.8	3.5	74.3	3.4	72.8
**BbkF**	**51.4**	**376.6**	**48.4**	**354.1**	**28.0**	**379.6**	**42.4**	**854.9**	**49.9**	**894.5**	**72.0**	**866.6**
**BaP**	**3074.5**	**6416.5**	**2105.2**	**5594.9**	**5244.7**	**6512.7**	**2782.5**	**12362.4**	**2811.3**	**13677.9**	**20918.3**	**13496.8**
**DahA**	**710.8**	**5183.7**	**550.2**	**5125.9**	**2095.8**	**5299.1**	**1787.1**	**13090.5**	**325.7**	**12804.6**	**8003.2**	**12329.0**
**InP**	**71.8**	**332.6**	**75.2**	**318.7**	**1779.1**	**326.5**	**723.9**	**766.2**	**1140.2**	**780.5**	**105.5**	**755.3**
BghiP	5.6	38.0	4.8	35.5	1.4	38.2	n.d	82.1	2.2	87.4	4.0	84.9
1N-Pyr	144.0	39.8	2.7	27.3	0.7	15.7	4.9	39.6	5.8	39.2	7.1	39.5
Total(16)	4711.3	13238.7	3171.7	12034.6	9604.7	13365.1	5610.3	27759.1	4655.1	29058.8	29421.5	28244.4

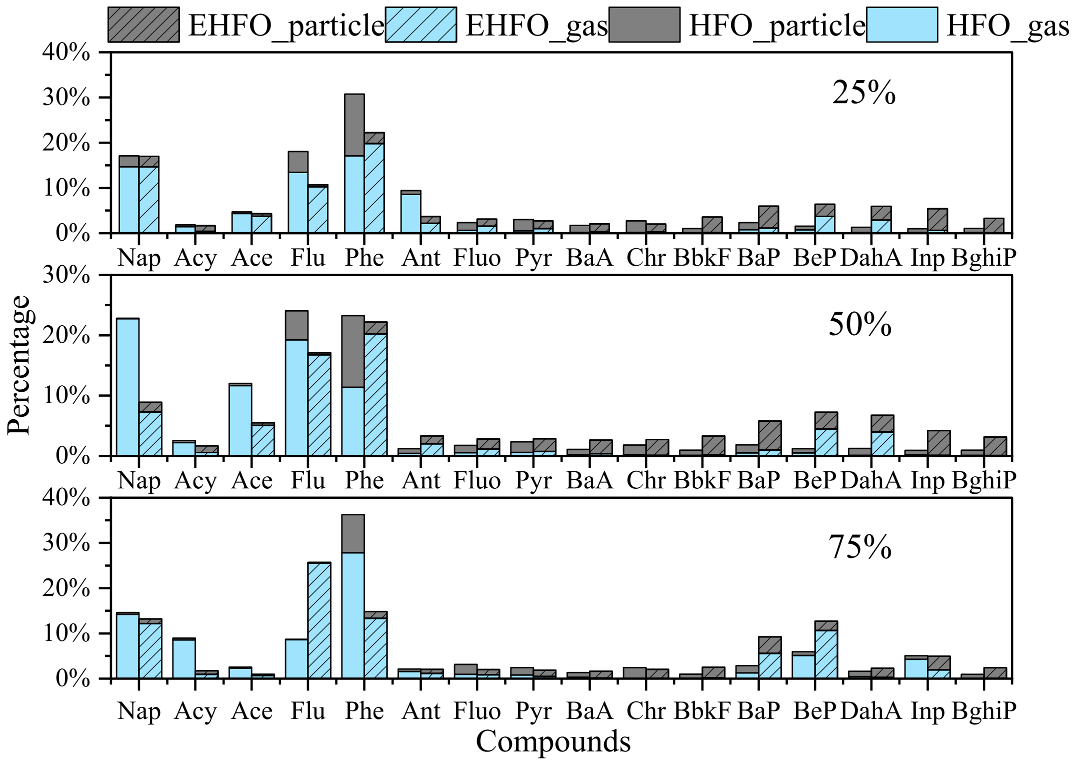

Figure S1. The distribution of PAH compounds in gaseous and particle phase.

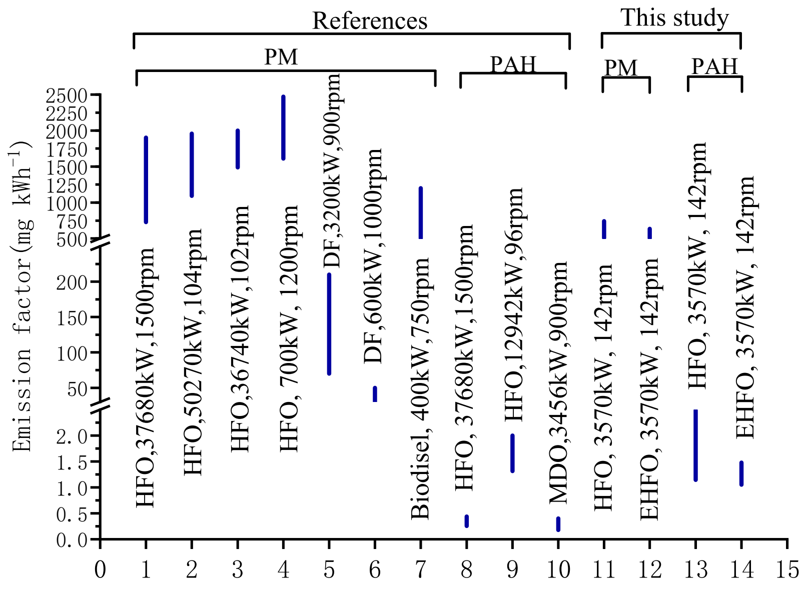

Figure S2. EFs of PM and PAHs in this study and references.

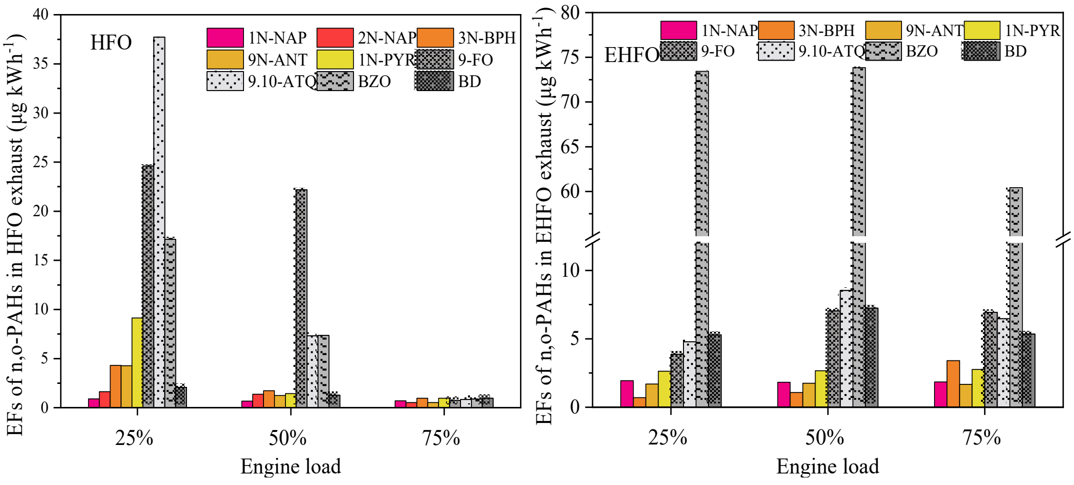

Figure S3. EFs of nPAHs and oPAHs.

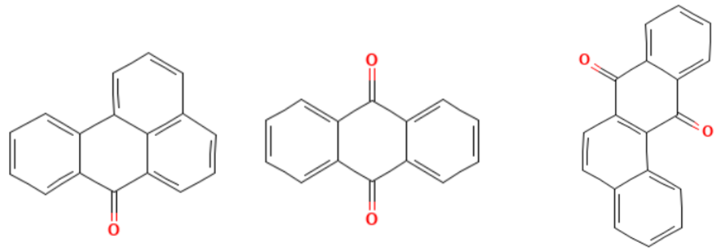

BZO 9,10-ATQ BD

Figure S4. The molecular structure of oPAHs.

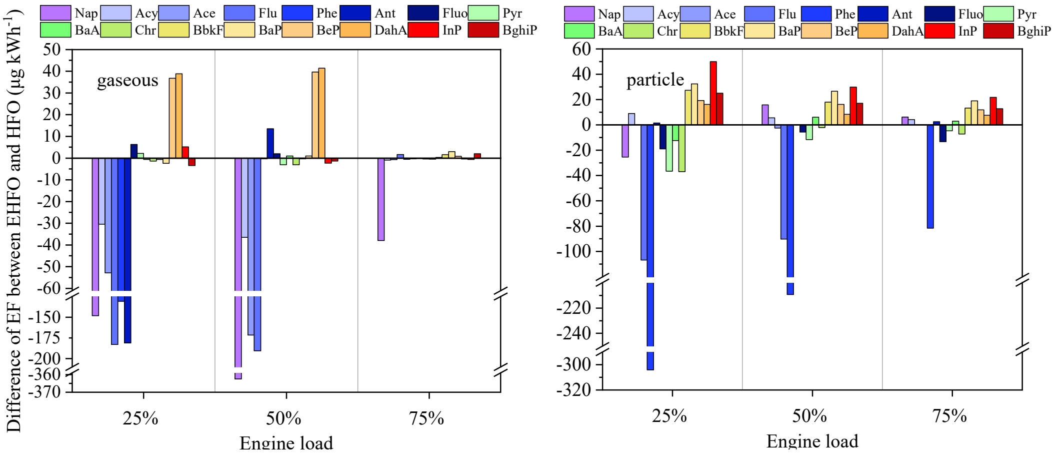

Figure S5. The difference in EFs between EHFO and HFO.

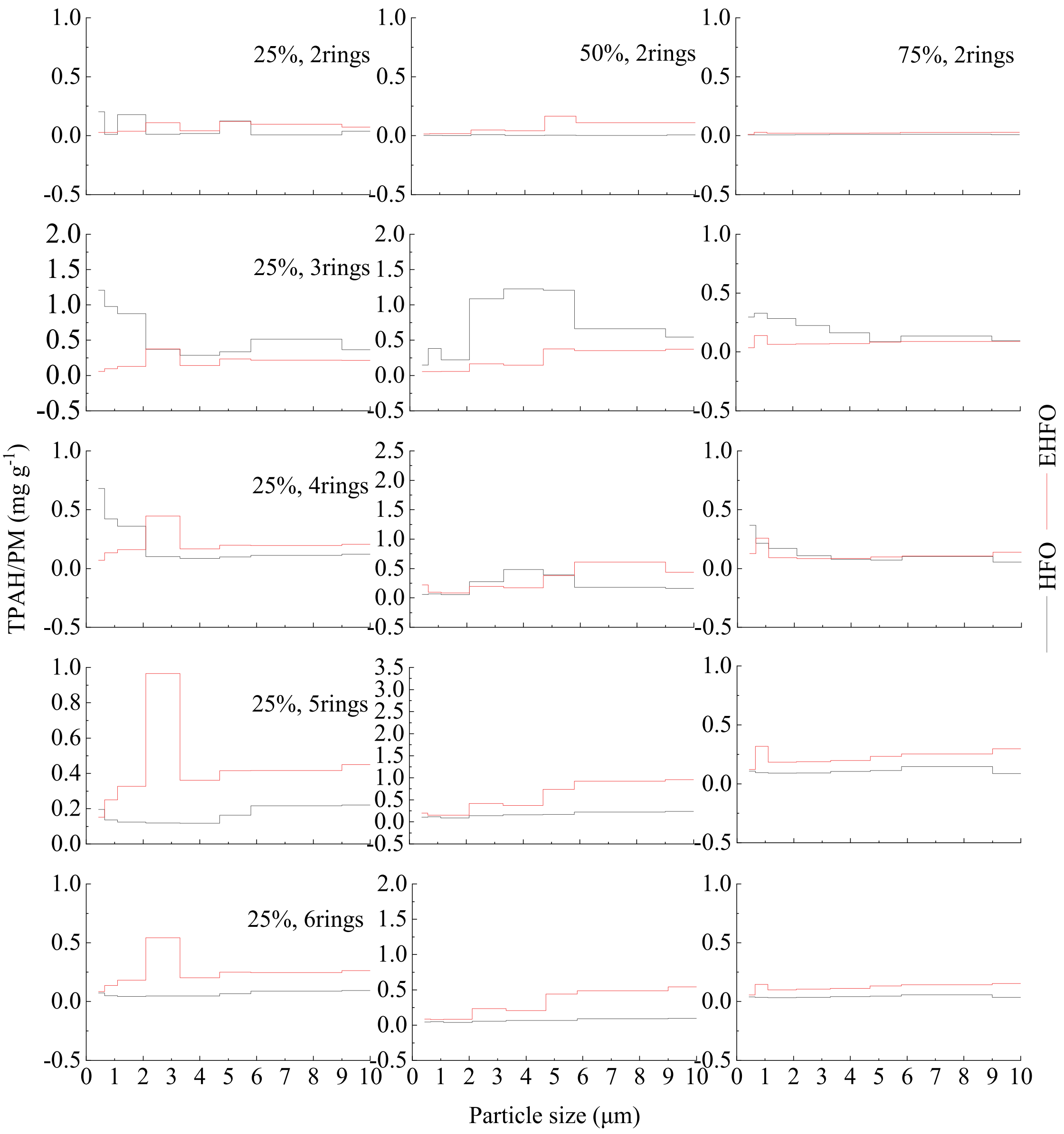

Figure S6. Particle-size distributions of PAH_{perPM}.

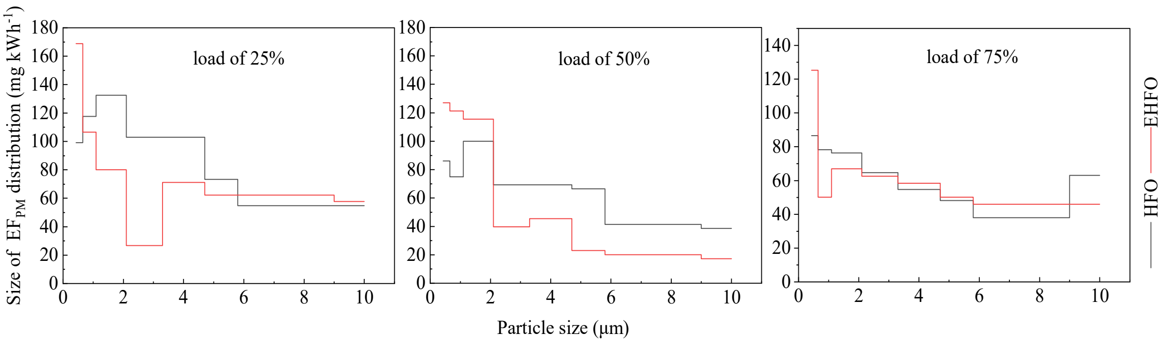

Figure S7. Size distribution of PM.

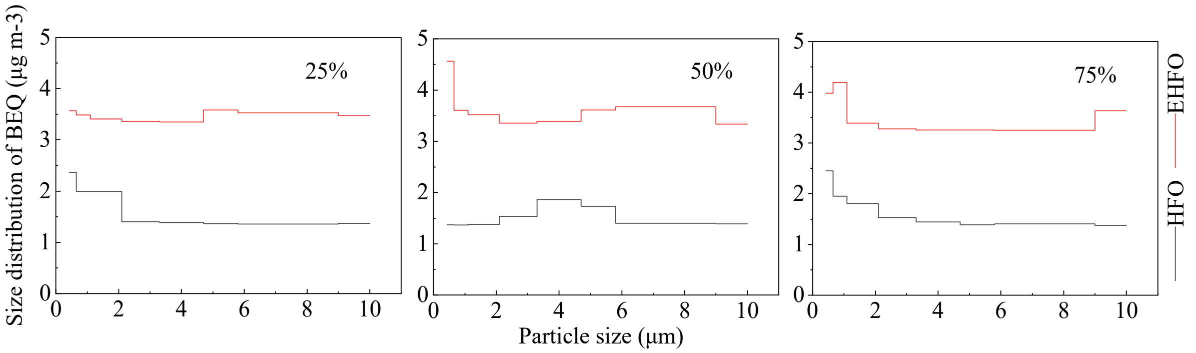

Figure S8. The $\sum BEQ$ in the particle phase.

References

1. Agrawal, H.; Malloy, Q.G.; Welch, W.A.; Miller, J.W.; Cocker, D.R. In-use gaseous and particulate matter emissions from a modern ocean going container vessel. *Atmospheric Environ.* **2008**, *42*, 5504–5510. https://doi.org/10.1016/j.atmosenv.2008.02.053.
2. Collins, J.F.; Brown, J.P.; Alexeeff, G.V.; Salmon, A.G. Potency Equivalency Factors for Some Polycyclic Aromatic Hydrocarbons and Polycyclic Aromatic Hydrocarbon Derivatives. *Regul. Toxicol. Pharmacol.* **1998**, *28*, 45–54.
3. Khan, M.Y.; Ranganathan, S.; Agrawal, H.; Welch, W.A.; Laroo, C.; Miller, J.W.; Cocker, D.R. Measuring in-use ship emissions with international and U.S. federal methods. *J. Air Waste Manag. Assoc.* **2012**, *63*, 284–291.
4. Nisbet, I.C.T.; Lagoy, P.K. Toxic equivalency factors (TEFs) for polycyclic aromatic hydrocarbons (PAHs). *Regul. Toxicol. Pharmacol.* **1992**, *16*, 290–300. https://doi.org/10.1016/0273-2300(92)90009-x.
5. Petzold, A.; Lauer, P.; Fritsche, U.; Hasselbach, J.; Lichtenstern, M.; Schlager, H.; Fleischer, F. Operation of Marine Diesel Engines on Biogenic Fuels: Modification of Emissions and Resulting Climate Effects. *Environ. Sci. Technol.* **2011**, *45*, 10394–10400. https://doi.org/10.1021/es2021439.
6. Sax, T.; Alexis, A. *A Critical Review of Ocean-Going Vessel Particulate Matter Emission Factors*; California Air Resource Board: Sacramento, CA, USA, 2007.
7. Sippula, O.; Stengel, B.; Sklorz, M.; Streibel, T.; Rabe, R.; Orasche, J.; Lintelmann, J.; Michalke, B.; Abbaszade, G.; Radischat, C.; et al. Particle Emissions from a Marine Engine: Chemical Composition and Aromatic Emission Profiles under Various Operating Conditions. *Environ. Sci. Technol.* **2014**, *48*, 11721–11729. https://doi.org/10.1021/es502484z.
8. van der Gon, H.D.; Hulskotte, J. *Methodologies for Estimating Shipping Emissions in The Netherlands: A Documentation of Currently Used Emission Factors and Related Activity Data*; Netherlands Environmental Assessment Agency: The Hague, The Netherlands, 2010.
9. Yu, H.; Duan, S.; Sun, P. Comparative analysis between natural gas/diesel (dual fuel) and pure diesel on the marine diesel engine. *J. Eng. Res.* **2015**, *3*, 37. https://doi.org/10.7603/s40632-015-0037-0.
10. Zhang, F.; Chen, Y.; Tian, C.; Lou, D.; Li, J.; Zhang, G.; Matthias, V. Emission factors for gaseous and particulate pollutants from offshore diesel engine vessels in China. *Atmospheric Meas. Technol.* **2016**, *16*, 6319–6334. https://doi.org/10.5194/acp-16-6319-2016.
11. Zhao, J.; Zhang, Y.; Chang, J.; Peng, S.; Hong, N.; Hu, J.; Lv, J.; Wang, T.; Mao, H. Emission characteristics and temporal variation of PAHs and their derivatives from an ocean-going cargo vessel. *Chemosphere* **2020**, *249*, 126194. https://doi.org/10.1016/j.chemosphere.2020.126194.
